# Better Screening Value of Sylvian Fissure Ratio on Cognitive Decline Among Female Compared to Male: An Observational Study in Elderly Patients With Cerebral Small Vessel Disease in Soochow

**DOI:** 10.3389/fnins.2021.729782

**Published:** 2021-10-04

**Authors:** Yiren Qin, Dannan Ai, Ashly E. Jordan, Xiaoning Guo, Tan Li, Shanshan Diao, Hongru Zhao, Yang Liu, Qun Xue, Yueju Wang, Qi Fang

**Affiliations:** ^1^The Stroke Center, The First Affiliated Hospital of Soochow University, Suzhou, China; ^2^Independent Researcher, New York, NY, United States; ^3^Department of Neurology, Saarland University, Homburg, Germany; ^4^Department of Geriatrics, The First Affiliated Hospital of Soochow University, Suzhou, China

**Keywords:** cognitive decline, sylvian fissure ratio, cerebral small vessel disease (CSVD), MoCA, females

## Abstract

**Background:** Cognitive decline (CD) occurs frequently in elderly patients with cerebral small vessel disease (CSVD). In China, elderly patients are more likely to enter healthcare in community hospitals where no magnetic resonance imaging (MRI) is available. This study aimed to explore the screening value of Sylvian fissure ratio (SFR) on CD and compare its gender difference from community-transferred patients.

**Methods:** We performed a single-center, observational study (collected between April 1, 2016, and March 1, 2019) to evaluate the association between Montreal Cognitive Assessment (MoCA) and SFR in 203 eligible community-transferred patients. Baseline characteristics of patients were collected during hospitalization. Multiple linear regression analyses were used to estimate the effect of variables on MoCA, and interactions between select variables were analyzed in different models. Receiver operating characteristic (ROC) curve analysis was used to evaluate the discriminative effect of SFR to severe CD.

**Results:** We identified that a meaningful SFR cutoff of 0.05 had important screening value (likelihood ratio test, *p* = 0.067) on CD. The ratio had a lower screen value in males when compared to females (adjusted β, −5.54; 95% CI, −8.78 to −2.30 vs. adjusted β, −1.01; 95% CI, −2.84 to 0.82). The gender difference was further verified by ROC curve analysis, in which this discriminative effect was more potent in females (from 0.878 to 0.948) compared to males (from 0.838 to 0.837).

**Conclusion:** An SFR of 0.05 may be more useful to distinguish CD in female patients with CSVD than male patients in whom the syndrome is suspected clinically.

## Introduction

Cerebral small vessel disease (CSVD) is a kind of cerebrovascular diseases which frequently co-occurs with the development of cognitive decline (CD). The burden of co-existing CSVD and CD is found in the elderly (Liu et al., 2019). However, the specific pathophysiological mechanisms of CD that accompany CSVD remains unknown (Liu et al., 2018). Additionally, the clinical manifestation of CD is nonspecific, there is a lack of objective diagnostic criteria, and there is notable heterogeneity of CD in patients with CSVD. Therefore, distinguishing vascular cognitive disorders (VCD) is a persisting challenge. To our knowledge, CSVD is an important subtype of vascular cognitive impairment (VCI) and is the most common cause of vascular dementia (Dichgans and Leys, 2017). Neuroimaging techniques provide tools for early diagnosis and may play an important role for cognitive endpoints in clinic (Zanon Zotin et al., 2021). The presentations of abnormalities on neuroimaging increased diagnostic accuracy (Tullberg et al., 2004; Sachdev P. et al., 2014). Therefore, not only latest international guidelines but recent Chinese guidelines which are relevant to VCD refer to its importance in identifying CD (Sachdev P. et al., 2014; Skrobot et al., 2018; Peng, 2019; Zhang et al., 2020). Consequently, identifying VCD may be highly dependent on neuroimaging techniques, which are not universally available in all Chinese healthcare settings (Chen et al., 2019).

In China, the use of magnetic resonance imaging (MRI) in regional central hospitals has become increasingly common in recent years, which has increased CSVD detection rates at the population-level (Wu et al., 2019). However, uneven distribution of neuroimaging resources exists in China over the non-central areas (i.e., non-polyclinic hospitals where shortage in professional neurologists and imaging equipment). At present, in China, a significant number of community/non-polyclinic doctors do not receive training on cognitive assessment tools (Zhuang et al., 2021). Moreover, in addition to the insufficient trained doctors, the poor compliance and inner resistance for diagnosis of dementia among the patients or their families may also underlie the low detection rate of mild cognitive impairment (MCI). However, a higher prevalence of MCI in the rural than in the urban population has been reported in China (Wang et al., 2011; Jia et al., 2014). Therefore, it is common that with low scores on cognitive screening tools such as MoCA, the patients still remain undetected by the healthcare system in China. In those non-central areas, non-contrast computerized tomography (CT) is the most common imaging technique available in their hospitals. Of patients with ischemic stroke, approximately 90% of patients receive brain CT, whereas only about 50% of patients receive MRI in China (Huang et al., 2010). Using linear measurements based either on CT or MRI could provide useful prognostic information eliminating the need for specialized software (Wardlaw et al., 2013). Among these approaches, the use of a parameter Sylvian fissure ratio (SFR) which represents cerebral atrophy is recommended. SFR is defined as the average maximum width of Sylvian fissures divided by the transpineal inner table diameter (Gomori et al., 1984; van Zagten et al., 1999). There is potential for SFR to predict outcomes in the 90-day functional profile of patients with moderate-volume basal ganglia hemorrhage (Sato et al., 2016; Kwon et al., 2018).

Until now, the screening value of SFR on CD in CSVD patients has not been fully examined. This study aimed to investigate its association with CD in patients with CSVD and compare the gender difference in the screening value of SFR.

## Materials and Methods

### Study Populations

Between April 1, 2016, and March 1, 2019, an inpatient-based observational cohort study was conducted in the Stroke Center, at the First Affiliated Hospital of Soochow University. A total of 203 eligible community-transferred patients (mean age 82.24 ± 4.91 years) were enrolled in this study. The inclusion criteria were (1) age equal to or greater than 65 years; (2) suspected presence of CSVD with cognitive impairment assessed by community doctors; (3) receipt of comprehensive clinical evaluation that included a detailed medical history, clinical diagnoses, laboratory tests, and screen testing with a standardized test battery; and (4) receipt of an MRI scan of the brain. The following patients were excluded: (1) cognitive impairment diagnosed with clear etiology, such as poisoning, infection, degeneration disease (Parkinson’s disease, multiple system atrophy, corticobasal degeneration, and dementia with Lewy bodies, etc.), immune demyelination (multiple sclerosis, Balo’s concentric sclerosis, etc.); (2) patients with intracranial tumors, large cerebral infraction, hydrocephalus; and (3) patients with visual or hearing impairment or language barrier which may affect their participation in the test or psychiatric symptoms with definite etiology. The ethics committees of The First Affiliated Hospital of Soochow University approved this study, which has been performed in accordance with the ethical standards laid down in the 1964 Declaration of Helsinki.

### Cognitive Assessment and Image Analysis

Montreal Cognitive Assessment (MoCA) was used to evaluate the cognitive functioning of the study population (Davis et al., 2015). The raw MoCA score was adjusted for educational attainment (1 extra point for 10–12 years of formal education; 2 points added for 4–9 years of formal education). Severe CD was determined by MoCA scores of ≤20 and were calculated using spline regression (see Table 1). All scales were evaluated by well-trained physicians independently blinded to the patient’s clinical details. Imaging of the brain was performed with a 3.0 T MRI scanner which included axial T1-weighted, axial T2-weighted, susceptibility-weighted, and fluid attenuated inversion recovery (FLAIR). White matter lesions were evaluated in four grades from the T2-weighted and FLAIR MR images by the supervision of two neuroradiologists. The differences between two neuroradiologists were solved by a senior consensus, according to the modified Fazekas visual scale (Fazekas et al., 1987). Briefly, Grade 0 was defined as No lesions; Grade 1: Mild lesions defined as pencil-like or cap-like thin lesions; Grade 2: Moderate lesions defined as smooth haloes; and Grade 3: Severe lesions defined as large fused lesions (Caprio et al., 2013). SFR was defined as the average of the maximal Sylvian widths taken from the cut showing the widest Sylvian fissure divided by the transpineal coronal inner table diameter (van Zagten et al., 1999). We adopted the criteria for the diagnosis of CSVD as STRIVE Recommendation mentioned (Wardlaw et al., 2013). The diagnosis of CSVD contains at least one of the following neuroimaging markers: (1) Lacunar infarction includes recent small subcortical infarct (RSSI) and lacune of presumed vascular origin. RSSI refers to recent infarction in the territory supplied by a perforating arteriole, which is less than 20 mm in the axial plane in its maximum diameter. The occurrence of lesion is consistent with imaging features or clinical syndrome in the previous few weeks. Lacune of presumed vascular origin was defined as a CSF-filled cavity (3–15 mm in diameter, with CSF similar signal on all sequences) surrounded by a high signal rim on T2-FLAIR. (2) White matter hyperintensity of presumed vascular origin is defined as punctate, patchy, or confluent hyperintense signal on T2-weighted and FLAIR sequences, and isointensity or hypointensity signal on T1-weighted sequence. The hyperintense signal in subcortical gray matter and brainstem is not included. (3) Cerebral microbleed is defined as small hypointensity signal with diameter ranging from 2 to 10 mm on susceptibility weighted imaging but no corresponding signal on FLAIR, T1-weighted, or T2-weighted sequences. (4) Perivascular space is defined as round (axial) or linear (parallel to vessels) fluid-filled space with diameter of less than 3 mm. The signal is similar to CSF that is hypointense on T1-weighted and hyperintense on T2-weighted sequence without hyperintense rim on T2-weighted or FLAIR sequence. We omit the definition of brain atrophy in our manuscript due to lack of specificity of atrophy and selection bias to our study. The assessment of SFR was defined as follows: based on the previous researches (Gomori et al., 1984; van Zagten et al., 1999), SFR was defined as the average of the maximal Sylvian widths taken from the cut showing the widest Sylvian fissure divided by the transpineal coronal inner table diameter.

**TABLE 1:** Threshold effect of SFR on MoCA using two-piecewise linear regression.

	β (95% CI) *p* value	LR test *p* value	Predicted MoCA at threshold (95% CI)
***Total***		0.067	20.04 (18.75, 21.32)
SFR ≤ 0.05	−109.37 (−185.97, −32.77) 0.0056		
SFR > 0.05	−28.86 (−48.88, −8.83) 0.0052		
***Male***		0.272	20.23 (18.70, 21.76)
SFR ≤ 0.05	−92.18 (−186.39, 2.03) 0.0571		
SFR > 0.05	−33.55 (−56.86, −10.23) 0.0055		
***Female***		0.047	19.25 (16.85, 21.64)
SFR ≤ 0.05	−162.18 (−290.93, −33.43) 0.0169		
SFR > 0.05	−10.04 (−49.50, 29.41) 0.6200		

### Data Collection

From the clinical dataset between April 1, 2016, and March 1, 2019, we obtained the following data elements for each patient: age; sex; Fazekas scale; and past and current diagnoses, such as hypertension, diabetes, coronary heart disease, stroke; patient-reported laboratory tests, such as creatinine, uric acid, α-hydroxybutyrate dehydrogenase (α-HBDH), high-sensitivity C-reactive protein, total cholesterol, total triglyceride, high-density lipoprotein cholesterol (HDLC), low-density lipoprotein cholesterol, apolipoprotein A (APOA), and apolipoprotein B.

### Statistical Analysis

Continuous variables were expressed as mean ± standard deviation (SD) when data accorded with normal distribution. Otherwise, data were expressed in terms of quartile. Data were compared using an unpaired, two-tailed *t*-test or Mann–Whitney *U* test. Categorical variables were compared using χ^2^ test or Fisher’s exact test. Furthermore, to help account for the nonrandomized allocation of SFR, we performed propensity-score methods to reduce the effects of potential confounding. A two-piecewise linear regression model was used to examine the threshold effect of SFR on MoCA through spline smoothing. We also applied a likelihood model to compare the one-linear regression model with a two-piecewise regression model. The βs and 95% CIs of MoCA in response to dichotomous SFR categorical variables across sex stratification were estimated, and their interactions were analyzed. Multiple linear regression analyses were also used to estimate the effect of variables on MoCA, with the lower dichotomous as the reference. The βs and 95% CIs were estimated by three kinds of adjusted models. The final model retained selected factors to develop an integrative predictive model to discriminate patients with severe CD (MoCA ≤ 20). The 95% CI with the area under the curve (AUC) was estimated using the bootstrapping method (500 iterations). The image of scatter plots with the best linear fit, together with the shadows up and down showing the 95% CI, was processed using MATLAB R2017b (version 9.3.0.713579; The Mathworks, Inc., Natick, MA, United States). All analyses were performed using the statistical package R version 3.6.3^1^.

## Results

### Association Between Montreal Cognitive Assessment Score and Sylvian Fissure Ratio

**FIGURE 1:**
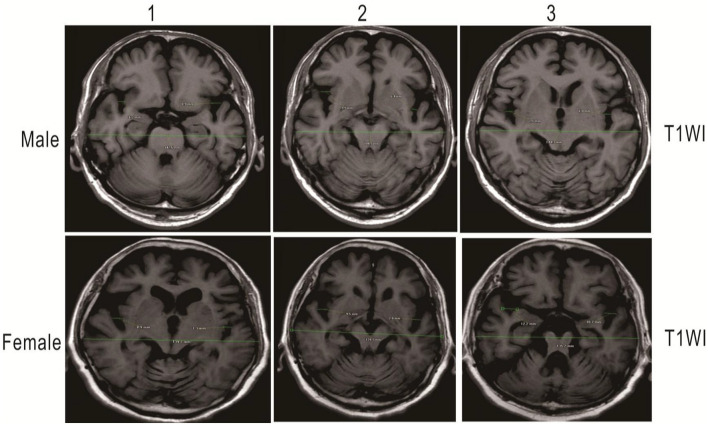
As a measure of external cerebral atrophy, SFR, the average of the maximum width of the two Sylvian fissures on the section showing them at their widest, divided by the transpineal coronal inner table diameter. Illustrative measurement of brain MRI images showing green lines in female and male patients, respectively. Measurement values are automatically generated by imaging software. Upper: A 73-year-old man with SFR 0.051: MoCA score: 20 on T1WI. Lower: A 76-year-old woman with SFR 0.068: MoCA score: 14 on T1WI. T1WI, T1 weighted image.

**FIGURE 2:**
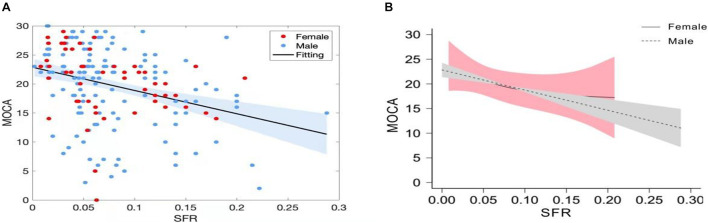
**(A)** Sylvian fissure ratio (SFR) was inversely associated with MoCA score. Scatter plots with the best linear fit were presented with the shadows up and down showing the 95% CI. Red dots represent female patients and blue dots represent male patients. **(B)** Smoothing spline plots of MoCA by SFR in stratified patients. Dashed line with gray shadows showing the 95% CI represents male patients, and solid line with pink shadows showing the 95% CI represents female patients.

**FIGURE 3:**
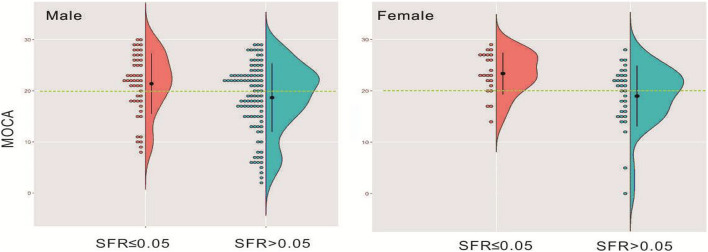
Distribution of MoCA in gender-stratified patients who were divided into dichotomous SFR illustrated in the half-violin plots with raw values.

A total of 203 patients who received a screening test (MoCA) were enrolled in the study. The detailed information on the demographic characteristics of the sample before analysis was shown in Supplementary Table 1. The illustrative brain MRI images with a continuous range of SFR measurements are provided in Figure 1. Scatter plots of raw values are shown in Figure 2A, and the regression was presented with the best linear fit plotted in a continuous line, with shadows up and down showing the 95% CI. When analyzed as continuous variables, the linear regression model showed that SFR was inversely associated with the MoCA score (Figure 2A). Smoothing spline plots suggested that an SFR score of 0.05 may represent a threshold to reliably distinguish the two-piecewise effect of SFR on MoCA (likelihood ratio test, *p* = 0.067) which corresponded to the predictive MoCA score of 20.04 (95% CI 18.75–21.32; Table 1). The two-piecewise effect of SFR was statistically significant in females, compared to males (Figure 2B and Table 1, likelihood ratio test, *p* = 0.047 vs. 0.272). The figures (half-violin plots with raw values) of MoCA in different cohorts that were divided into dichotomous SFR are shown in Figure 3.

### Characteristics of the Study Population

The cohort was divided into dichotomous SFR categorical variables according to the threshold, that is, those with SFR of greater than 0.05 and less than or equal to 0.05 comprised the study groups, respectively. Of these 203 unmatched patients, 132 (65%) had an SFR greater than 0.05, while 71 had no more than 0.05. The unmatched cohort was propensity score-matched (1:1) for age and gender. The distribution of the cohort’s baseline characteristics according to SFR categorical variables is shown in Table 2 and Supplementary Table 2. Patients with SFR greater than 0.05 had older age, lower MoCA, lower APOA, and higher Fazekas scale than patients whose SFR was ≤0.05 (*p* < 0.05). In propensity score-matched analytic patients, statistical differences were found among age, MoCA, HDLC, and Fazekas scale (*p* < 0.05).

**TABLE 2:** Characteristics of patients divided into dichotomous SFR categorical variables before matching.

Characteristic	Mean (SD) or median (Q1–Q3)/N (%)		*p*-Value
	
	SFR ≤ 0.05 (*n* = 71)	SFR > 0.05 (*n* = 132)	
Age	80.75 ± 4.28	83.04 ± 5.06	0.001
MoCA	22.00 ± 5.43	18.49 ± 6.42	<0.001
SFR	0.03 ± 0.01	0.10 ± 0.05	<0.001
Creatinine	88.42 ± 36.93	84.65 ± 34.95	0.473
Uric acid	347.27 ± 94.33	350.52 ± 116.81	0.841
α-HBDH	151.09 ± 38.55	149.61 ± 33.70	0.778
HSCRP	3.43 ± 4.17	4.39 ± 11.95	0.514
TC	4.25 ± 0.80	4.16 ± 0.89	0.471
TG	1.25 ± 0.61	1.44 ± 0.83	0.094
HDLC	1.24 ± 0.32	1.17 ± 0.32	0.137
LDLC	2.50 ± 0.64	2.44 ± 0.74	0.563
APOA	1.39 (1.21–1.63)	1.29 (1.12–1.49)	0.005
APOB	0.90 ± 0.22	0.89 ± 0.24	0.688
***Fazekas scale, No. (%)***			0.002
0	29 (40.8)	26 (19.7)	
1	22 (31)	36 (27.3)	
2	15 (21.1)	45 (34.1)	
3	5 (7)	25 (18.9)	
***Sex-No. (%)***			0.300
Male	49 (69)	100 (75.8)	
Female	22 (31)	32 (24.2)	
***Hypertension, No. (%)***			0.301
No	8 (11.3)	22 (16.7)	
Yes	63 (88.7)	110 (83.3)	
***Diabetes, No. (%)***			0.087
No	47 (66.2)	71 (53.8)	
Yes	24 (33.8)	61 (46.2)	
***Coronary heart disease, No. (%)***			0.956
No	53 (74.6)	99 (75)	
Yes	18 (25.4)	33 (25)	
***Stroke, No. (%)***			0.269
No	60 (84.5)	103 (78)	
Yes	11 (15.5)	29 (22)	

*MoCA denotes, Montreal Cognitive Assessment; SFR, Sylvian fissure ratio; α-HBDH, α-hydroxybutyrate dehydrogenase; HSCRP, high-sensitivity C-reactive protein; TC, total cholesterol; TG, total triglyceride; HDLC, high-density lipoprotein cholesterol; LDLC, low-density lipoprotein cholesterol; APOA, apolipoprotein A; and APOB, apolipoprotein B.*

### Associations of Montreal Cognitive Assessment and Sylvian Fissure Ratio Categorical Variables by Subgroup Analysis

The regression coefficients (95% CI) for the association between MoCA and SFR categorical variables in the stratified cohort were explored. As shown in Table 3, interaction effects were also analyzed among the stratum of sex. There was a statistically significant modification effect of sex on the associations of MoCA with SFR categorical variables (*p* = 0.0114 and *p* = 0.0129, respectively) in the two adjusted models.

**TABLE 3:** Modification effect of gender on the association between MoCA and SFR categorical variables.

	β (95% CI) *p* value		
	
	Non-adjusted	Adjust I	Adjust II
***Total***			
0.03 ± 0.01	Ref.	Ref.	Ref.
0.10 ± 0.05	−3.49 (−5.25, −1.72) 0.0001	−1.89 (−3.45, −0.32) 0.0190	−1.70 (−3.27, −0.14) 0.0344
***Male***			
0.03 ± 0.01	Ref.	Ref.	Ref.
0.10 ± 0.05	−2.72 (−4.92, −0.52) 0.0168	−0.92 (−2.75, 0.90) 0.3218	−1.01 (−2.84, 0.82) 0.2808
***Female***			
0.03 ± 0.01	Ref.	Ref.	Ref.
0.10 ± 0.04	−5.43 (−8.13, −2.72) 0.0003	−5.80 (−8.72, −2.88) 0.0003	−5.54 (−8.78, −2.30) 0.0020
***P for interaction***	0.1713	0.0114	0.0129

*Adjusted model 1 was adjusted for risk factors of CSVD (Fazekas; age; hypertension; diabetes; coronary heart disease; and stroke). Adjusted model 2 was adjusted for all covariables. SFR denotes Sylvian fissure ratio.*

### Linear Regression Analyses and Development of a Model Assessing Cognition

**FIGURE 4:**
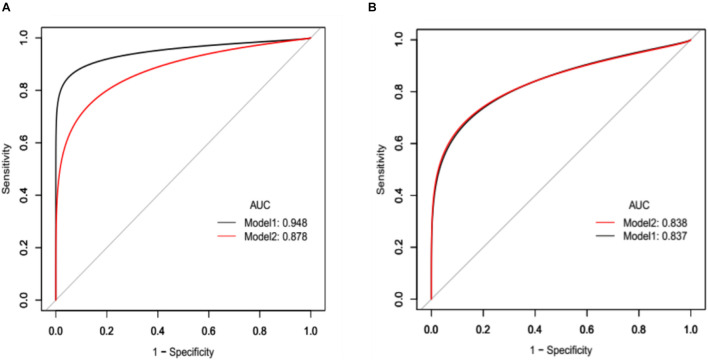
**(A)** Receiver operating characteristic (ROC) curve analyses of integrative variables (Fazekas scale, having stroke, SFR categorical variables, and age) to discriminate female patients with severe cognitive impairment. Area under the ROC curve (AUC) for model 1, SFR rule in was 0.9477 (95% CI, 0.8115–0.9730), and for model 2, SFR rule out was 0.8775 (95% CI 0.7490–0.9466). **(B)** ROC analyses of integrative variables (Fazekas scale, having stroke, SFR categorical variables, and age) to discriminate male patients with severe cognitive impairment. AUC for model 1, SFR rule in was 0.8367 (95% CI, 0.7662–0.8941), and for model 2, SFR rule out was 0.8380 (95% CI 0.7585–0.8899).

Three regression models were analyzed for crude adjustment for risk factors of CSVD including Fazekas; age; hypertension; diabetes; coronary heart disease; and stroke; the corresponding βs (95% CI) are depicted in Tables 4, 5. Having higher Fazekas scale scores, experiencing stroke in both male (Table 4) and female patients (Table 5), and having SFR > 0.05 were statistically and clinically significant. The factors of Fazekas scale, occurrence of stroke, SFR threshold (0.05), and age were retained in the final analysis to develop an integrative predictive model to discriminate patients with severe CD (MoCA ≤ 20). Two Receiver operating characteristic models adjusted for the resulting factors (model 1, SFR rule in, and model 2, SFR rule out), with bootstrap validation, and were applied and compared (Figures 4A,B). The predictive performance was improved as evidenced by a higher AUC in female patients (0.878 vs. 0.948), whereas an improvement was not observed in male patients (0.838 vs. 0.837).

**TABLE 4:** Effect of variables on MoCA in three linear regression models among male patients.

Variable	β (95%CI) *p*-Value		
	
	Non-adjusted	Adjust I	Adjust II
***Age***	−0.24 (−0.46, −0.03) 0.0295	−0.11 (−0.29, 0.06) 0.1932	−0.10 (−0.29, 0.09) 0.2880
***Fazekas***			
1	−2.72 (−5.00, −0.45) 0.0203	−2.67 (−4.91, −0.43) 0.0211	−2.76 (−5.04, −0.49) 0.0188
2	−9.00 (−11.29, −6.72) <0.0001	−8.21 (−10.54, −5.88) <0.0001	−8.49 (−10.85, −6.13) <0.0001
3	−10.07 (−12.67, −7.46) <0.0001	−9.19 (−11.87, −6.51) <0.0001	−9.34 (−12.08, −6.60) <0.0001
***HBP***	−3.76 (−6.61, −0.90) 0.0110	−1.60 (−3.93, 0.73) 0.1792	−1.22 (−3.60, 1.15) 0.3151
***Diabetes***	0.19 (−1.97, 2.35) 0.8638	1.48 (−0.21, 3.17) 0.0891	1.54 (−0.25, 3.34) 0.0943
***CHD***	−0.51 (−2.93, 1.91) 0.6803	−0.79 (−2.67, 1.09) 0.4131	−0.80 (−2.89, 1.28) 0.4526
***Stroke***	−4.98 (−7.42, −2.54) 0.0001	−2.95 (−4.98, −0.92) 0.0051	−2.98 (−5.10, −0.87) 0.0064
***SFR*** >0.05	−2.72 (−4.92, −0.52) 0.0168	−0.92 (−2.75, 0.90) 0.3218	−1.01 (−2.84, 0.82) 0.2808
***Creatinine*** > = 111	0.89 (−1.84, 3.63) 0.5224	1.88 (−0.42, 4.17) 0.1112	2.48 (−0.79, 5.75) 0.1392
***Uric acid*** > = 428	−0.81 (−3.30, 1.67) 0.5231	−0.02 (−2.03, 1.98) 0.9820	0.82 (−2.14, 3.79) 0.5875
***α−HBDH*** > = 182	0.02 (−3.15, 3.18) 0.9914	0.26 (−2.23, 2.76) 0.8368	−0.89 (−4.71, 2.93) 0.6501
***HSCRP*** > = 3	0.08 (−2.19, 2.35) 0.9465	0.72 (−1.03, 2.47) 0.4221	0.52 (−1.48, 2.51) 0.6131
***TC*** > = 5.2	−1.70 (−5.57, 2.17) 0.3901	−1.32 (−4.35, 1.72) 0.3966	−2.40 (−6.35, 1.56) 0.2370
***TG*** > = 1.7	0.60 (−1.94, 3.14) 0.6443	0.42 (−1.57, 2.42) 0.6773	−0.76 (−4.21, 2.69) 0.6681
***HDLC*** > = 1	0.85 (−1.38, 3.08) 0.4570	−0.35 (−2.18, 1.49) 0.7124	−0.63 (−3.07, 1.81) 0.6153
***LDLC*** > = 3.4	−0.12 (−4.15, 3.92) 0.9544	−0.34 (−3.50, 2.81) 0.8321	−1.32 (−5.37, 2.73) 0.5251
***APOA*** > = 1.6	3.31 (0.54, 6.08) 0.0205	2.16 (−0.05, 4.38) 0.0579	1.34 (−1.26, 3.94) 0.3135
***APOB*** > = 1.25	2.71 (−2.64, 8.06) 0.3219	4.62 (0.51, 8.72) 0.0290	2.51 (−3.17, 8.19) 0.3889

*Adjusted model 1 was adjusted for risk factors of CSVD (Fazekas; age; hypertension; diabetes; coronary heart disease; and stroke). Adjusted model 2 was adjusted for all covariables.*

*HBP, hypertension; CHD, coronary heart disease; SFR, Sylvian fissure ratio; α-HBDH, α-hydroxybutyrate dehydrogenase; HSCRP, high-sensitivity C-reactive protein; TC, total cholesterol; TG, total triglyceride; HDLC, high-density lipoprotein cholesterol; LDLC, low-density lipoprotein cholesterol; APOA, apolipoprotein A; and APOB, apolipoprotein B.*

**TABLE 5:** Effect of variables on MoCA in three linear regression models among female patients.

Variable	β (95% CI) *p*-Value		
	
	Non-adjusted	Adjust I	Adjust II
***Age***	−0.30 (−0.59, −0.01) 0.0482	−0.22 (−0.51, 0.08) 0.1604	−0.01 (−0.40, 0.37) 0.9397
***Fazekas***			
1	0.44 (−3.34, 4.21) 0.8218	1.41 (−2.30, 5.11) 0.4603	1.54 (−2.60, 5.68) 0.4699
2	−3.48 (−7.06, 0.10) 0.0623	−1.72 (−5.36, 1.92) 0.3599	−2.53 (−7.40, 2.33) 0.3145
3	−3.92 (−9.82, 1.97) 0.1983	−0.34 (−6.72, 6.04) 0.9179	−3.10 (−10.07, 3.86) 0.3887
***HBP***	−2.78 (−7.23, 1.66) 0.2251	−1.33 (−5.78, 3.11) 0.5592	−0.26 (−4.96, 4.45) 0.9159
***Diabetes***	−0.80 (−3.83, 2.22) 0.6040	0.63 (−2.34, 3.61) 0.6781	−1.71 (−4.94, 1.52) 0.3057
***CHD***	−1.82 (−5.32, 1.69) 0.3150	−0.55 (−4.10, 2.99) 0.7614	−1.85 (−5.61, 1.91) 0.3421
***Stroke***	−6.34 (−10.24, −2.44) 0.0024	−5.35 (−9.95, −0.74) 0.0276	−4.29 (−9.16, 0.58) 0.0930
***SFR*** >0.05	−5.43 (−8.13, −2.72) 0.0003	−5.80 (−8.72, −2.88) 0.0003	−5.54 (−8.78, −2.30) 0.0020
***Creatinine*** > = 111	4.43 (−2.07, 10.93) 0.1874	5.05 (−1.38, 11.47) 0.1309	−3.14 (−14.19, 7.90) 0.5804
***Uric acid*** > = 428	−1.29 (−5.17, 2.60) 0.5190	−0.67 (−4.68, 3.35) 0.7463	−3.32 (−10.13, 3.49) 0.3460
***α−HBDH*** > = 182	−0.88 (−4.32, 2.57) 0.6210	−0.25 (−3.76, 3.25) 0.8881	1.25 (−4.59, 7.09) 0.6777
***HSCRP*** > = 3	−1.05 (−4.68, 2.58) 0.5743	−1.57 (−5.17, 2.02) 0.3959	−2.39 (−9.89, 5.11) 0.5368
***TC*** > = 5.2	−0.41 (−4.17, 3.34) 0.8298	−1.08 (−4.91, 2.75) 0.5829	0.44 (−5.59, 6.47) 0.8876
***TG*** > = 1.7	0.47 (−2.98, 3.93) 0.7886	−0.37 (−3.85, 3.12) 0.8373	−4.02 (−11.05, 3.00) 0.2691
***HDLC*** > = 1	0.67 (−3.22, 4.57) 0.7363	0.11 (−3.79, 4.01) 0.9547	0.29 (−5.17, 5.75) 0.9178
***LDLC*** > = 3.4	−0.83 (−5.33, 3.68) 0.7204	−1.77 (−6.21, 2.66) 0.4377	−4.93 (−13.16, 3.30) 0.2487
***APOA*** > = 1.6	−0.57 (−3.95, 2.80) 0.7403	−0.34 (−3.97, 3.29) 0.8554	−2.42 (−8.33, 3.49) 0.4284
***APOB*** > = 1.25	0.58 (−4.23, 5.40) 0.8133	−0.44 (−5.22, 4.34) 0.8574	−3.95 (−10.91, 3.00) 0.2728

*Adjusted model 1 was adjusted for risk factors of CSVD (Fazekas; age; hypertension; diabetes; coronary heart disease; stroke). Adjusted model 2 was adjusted for all covariables.*

*HBP, hypertension; CHD, coronary heart disease; SFR, Sylvian fissure ratio; α-HBDH, α-hydroxybutyrate dehydrogenase; HSCRP, high-sensitivity C-reactive protein; TC, total cholesterol; TG, total triglyceride; HDLC, high-density lipoprotein cholesterol; LDLC, low-density lipoprotein cholesterol; APOA, apolipoprotein A; and APOB, apolipoprotein B.*

## Discussion

Our analysis identified a clinically significant value of 0.05 for the SFR in that this threshold was specifically useful as a screen predictor of CD in female patients with CSVD (Adjusted β, −5.54; 95% CI, −8.78 to −2.30).

Cerebral small vessel disease patients with manifesting latent and chronic CD is very common. The type and degree of CD is related to the neuroimaging features, the number of lesions, and the location of lesions (Cannistraro et al., 2019). Thus, the management of its diagnostic approaches needs to be further improved in order to improve outcomes among these patients (Teng et al., 2017; Smith and Beaudin, 2018). Traditionally, CD is primarily assessed by screening tools, such as Mini-Mental State Examination (MMSE), MoCA, Addenbrooke’s Cognitive Examination-Revised, etc. Compared to other screen tools, MoCA has many advantages: (1) It has higher sensitivity for detecting MCI and is superior for the detection of post-stroke cognitive impairment (PSCI; Dong et al., 2010; Pendlebury et al., 2012). (2) Although specificity of the MoCA is slightly lower than the MMSE, it still performs good (87%; Nasreddine et al., 2005; Shi et al., 2018). (3) Since 2006, MoCA has been recommended as a clinical screening instrument for VCI by the NINDS-CSN working group (Hachinski et al., 2006). It is valid and reliable in the patients with VCI and CSVD and has good psychometric properties across a wide range of VCI-related conditions and is free for clinical and research use (Wong et al., 2009, 2013). Different from the MMSE, MoCA covers most of the domains considered typically affected in cerebrovascular diseases (executive functions, attention, and concentration) and has been extensively applied in studies (particularly in Asia; Salvadori et al., 2020). (4) According to the latest guidelines (Jia et al., 2014; Skrobot et al., 2018), MoCA is better than MMSE in identifying mild VCI and can be applied to the early screening and overall cognitive assessment of suspected VCI (Koski, 2013). Nevertheless, the threshold of MoCA adopted in PSCI has not reached a consensus (Davis et al., 2015). Neuroimaging techniques provide tools for early diagnosis and may play an important markers in clinical practice (Zanon Zotin et al., 2021). The presentations of neuroimaging is critical to increase diagnostic inaccuracy in the subtypes of CSVD (Tullberg et al., 2004; Sachdev P. et al., 2014). Therefore, not only latest international guidelines but recent Chinese guidelines which are relevant to VCD refer to its importance in identifying CD (Sachdev P. et al., 2014; Skrobot et al., 2018; Peng, 2019; Zhang et al., 2020). Therefore, objectiveness, homogeneity, and patient compliance may be optimized by combing neuropsychiatric inventory and neuroimaging.

Thus far, it is also difficult to conduct widespread cognitive screening assessment in China, especially in non-polyclinic hospitals, primary care settings, and community clinics where there is a shortage of neuropsychiatric professionals. However, a higher prevalence of MCI in the rural than in the urban population has been reported in China (Wang et al., 2011; Jia et al., 2014). Moreover, in addition to the insufficient trained doctors, the poor compliance and inner resistance for diagnosis of dementia in the patients or their families may also underlie the low prevalence of MCI (US Preventive Services Task Force et al., 2020). At present, in China, neuroimaging, especially CT, is widely used in many non-polyclinic hospitals (Wang et al., 2017, 2019). Many elderly people will go to non-polyclinic hospitals for treatment because of dizziness, headache, limb weakness, and other symptoms due to its convenience when compared to regional center hospitals. At this time, it is more acceptable for patients and their families when they are suggested to receive a neuroimaging exam to preclude diseases such as cerebral infarction or hemorrhage (He et al., 2019; Wang et al., 2020). Linear measurement plays an important role in the screening of CD (Bermel et al., 2002; Del Brutto et al., 2018), especially in identifying cerebral atrophy, which has been implicated in the pathophysiology of CD. Previous studies have found that the volume of nucleus accumbens, amygdala, caudate nucleus, thalamus, and brain stem is closely related to cognition in stroke patients (Hilal et al., 2015). One study showed that of 393 patients with transient ischemic attack or suspected stroke, 169 patients had a medial temporal lobe atrophy finding that medial temporal lobe atrophy was significantly related to memory, naming, perception, executive ability, speed, and attention (Kebets et al., 2015). According to the STRIVE recommendation, the neuroimaging markers of cerebral atrophy are nonspecific (Wardlaw et al., 2013). In the indices of brain atrophy, the Sylvian fissure is an important structure of both pathophysiological and microsurgical significance (Balak, 2014). The SFR has been shown to be associated not only with clinical outcomes in patients with CSVD (Sato et al., 2016) or with basal ganglia hemorrhage (Kwon et al., 2018) but also with survival in elderly individuals after age 85 (Olesen et al., 2011). Concerning the overall significance, similar to stroke center referrals, physicians in non-polyclinic hospitals primarily screen suspected patients of CSVD with CD through neuroimaging, and send them to regional central hospitals where there is an abundance of neuropsychiatric professionals for further confirmation. The practical application of using the SFR may increase the diagnostic accuracy and screening rate under the current Chinese healthcare system. In addition, it will be of great benefit to find out comparatively objective and simple parameters with limited harms of screening (e.g., labeling people with dementia) in the elderly (US Preventive Services Task Force et al., 2020).

Our primary finding is, to our knowledge, the first to identify a clinically significant screening value of 0.05 for SFR as a threshold associated with CD in patients with CSVD, specifically in female patients. We speculated that due to the higher weight of visuospatial and execution in MoCA, the scores of female patients may be lower than male patients in the same stage and that this may have clinical significance (Munro et al., 2012; Laws et al., 2016). Females have been found to experience more rapid cerebral decline compared to male patients with respect to the development of MCI in Alzheimer’s disease (AD; Perneczky et al., 2007; Bramen et al., 2011; Giedd et al., 2012; Koolschijn and Crone, 2013). The proposed difference of downward trend is consistent with our finding. Additionally, estrogen has been shown to have a protective effect on the nervous system, and estrogen can regulate glucose transport, aerobic glycolysis and mitochondrial function, thus generating ATP in multiple brain regions (such as medial temporal lobe, cingulate gyrus, and frontal cortex) involved in cognitive function, providing energy for brain tissue (Munro et al., 2012; Mosconi et al., 2018). During menopause, estrogen level decrease, increasing the risk of AD in female. In our study, all female patients were over 65 years old and were in menopause. Other factors that may lead to sex differences are that the risk of AD in females with the apoE4 gene is three to four times higher than that in non-carriers; this risk in men with or without apoE4 gene is relatively low (Bertram and Tanzi, 2008). There may be confounding due to the fact that life expectancy among females is higher than males in China, and that AD is predominately present in those older than 65 years (Clarfield and Dwolatzky, 2013). To improve the generalizability of the conclusions drawn, we also performed additional analysis using public data from ADNI. It was found that the optimal threshold increased based on the cohort analysis of the ADNI database when compared to our cohort (data not shown). Meanwhile, with the same increase of SFR, females have been found to experience more rapid CD compared to male patients in MMSE scores. However, the gender difference was not as big as the cohort with MOCA screen testing. We speculate that this may be related to the different sensitivity between MMSE and MOCA.

Our study has several limitations. The data were validated with a single-center observational study, and the optimal threshold for SFR may differ when other confounders are controlled in randomized controlled trials. Therefore, inferences derived from our results may lack generalizability and maybe only applicable to specific populations similar to our study population. SFR thresholds require further study in both female and male populations in studies with larger sample sizes using randomized methods. According to the *Diagnostic and Statistical Manual of Mental Disorders* (*DSM-V*; Sachdev P.S. et al., 2014), diagnostic criteria for mild/major neurocognitive disorder encompass evidence of six key cognitive domains (complex attention, executive function, learning and memory, language, perceptual–motor, or social cognition), together with capacity for independence in everyday activities. In our study, we only use MoCA as a screening tool. However, MoCA is not a diagnostic criterion of CD. The association of SFR and tests for different domains in *DSM-V* should be performed to indicate the predictive power of SFR. In future studies, specific tests should be administered per cognitive domain according to *DSM-V*, and further detailed evaluations of cognitive impairment with each domain together with IADL, NPI, etc., should be further investigated. Other limitations of this study include missing data for some important variables, such as lack of regular follow-up after discharge, lack of further evaluation of CD and SFR after discharge, lack of patient genotype records, etc. Finally, the single-center design may limit the generalizability of this conclusion.

In conclusion, among patients undergoing CSVD, higher SFR may be highly associated with a greater risk of CD. A ratio of 0.05 may represent a cutoff point defining higher risk for female patients in whom the disorder is clinically suspected. SFR may be a useful, low-resource, and objective measurement to screen CSVD patients with CD, especially for female elderly in non-polyclinic hospitals such as community hospitals and rural clinics where there is a shortage of professional neurologists and imaging equipment.

## Data Availability Statement

The data that support the findings of this study are available from the corresponding author upon reasonable request.

## Ethics Statement

The studies involving human participants were reviewed and approved by The Ethics Committees of The First Affiliated Hospital of Soochow University. The patients/participants provided their written informed consent to participate in this study.

## Author Contributions

YQ and QX: study design. DA: data acquisition. XG and TL: statistical analysis. SD and QX: data supervisor. YQ and YL: the manuscript preparation. HZ and YW: language editing. YW: the manuscript review. AJ: the manuscript review and revision (second round). YQ and YW: funding support. All authors contributed to the article and approved the submitted version. QF: director of the Stroke Center has organized all authors to finish this project.

## Conflict of Interest

The authors declare that the research was conducted in the absence of any commercial or financial relationships that could be construed as a potential conflict of interest.

## Publisher’s Note

All claims expressed in this article are solely those of the authors and do not necessarily represent those of their affiliated organizations, or those of the publisher, the editors and the reviewers. Any product that may be evaluated in this article, or claim that may be made by its manufacturer, is not guaranteed or endorsed by the publisher.

## Footnotes

1
http://www.r-project.org/
