# Supplementary material for: Better Screening Value of Sylvian Fissure Ratio on Cognitive Decline Among Female Compared to Male: An Observational Study in Elderly Patients With Cerebral Small Vessel Disease in Soochow
Source: Front Neurosci. 2021 Oct 4;15:729782. doi: 10.3389/fnins.2021.729782 (PMC8524438; doi:10.3389/fnins.2021.729782)
Supplement: Supplementary file 1 [file Data_Sheet_1.docx]

| Supplemental Table 1. Characteristics of studied population before analysis. | | |
| --- | --- | --- |
| Characteristic | | Mean(SD), Median (Q1-Q3) / N(%) |
| **Age** | | 82.24 (4.91), 82.00 (78.00-86.00) |
| **MoCA** | | 19.88 (6.33), 21.00 (17.00-24.00) |
| **SFR** | | 0.08 (0.05), 0.06 (0.04-0.11) |
| **Creatinine** | | 85.97 (35.61), 77.50 (64.65-97.00) |
| **Uric Acid** | | 349.38 (109.25), 342.00 (270.50-414.50) |
| **α-HBDH** | | 150.13 (35.38), 145.00 (128.00-164.00) |
| **HSCRP** | | 4.05 (9.94), 1.70 (0.90-3.90) |
| **TC** | | 4.19 (0.86), 4.12 (3.55-4.76) |
| **TG** | | 1.38 (0.76), 1.17 (0.82-1.66) |
| **HDLC** | | 1.20 (0.32), 1.14 (0.94-1.38) |
| **LDLC** | | 2.46 (0.71), 2.43 (1.97-2.94) |
| **APOA** | | 1.40 (0.64), 1.31 (1.15-1.54) |
| **APOB** | | 0.89 (0.23), 0.88 (0.73-1.02) |
| **Fazekas scale-No. (%)** | | |
| 0 | 55 (27.09) | |
| 1 | 58 (28.57) | |
| 2 | 60 (29.56) | |
| 3 | 30 (14.78) | |
| ***Sex-No. (%)*** | | |
| male | 149 (73.40) | |
| female | 54 (26.60) | |
| ***Hypertension -No. (%)*** | | |
| No | 30 (14.78) | |
| Yes | 173 (85.22) | |
| ***Diabetes -No. (%)*** | | |
| No | 118 (58.13) | |
| Yes | 85 (41.87) | |
| ***Coronary Heart Disease-No. (%)*** | | |
| No | 152 (74.88) | |
| Yes | 51 (25.12) | |
| ***Stroke-No. (%)*** | | |
| No | 163 (80.30) | |
| Yes | 40 (19.70) | |

MoCA denotes Montreal Cognitive Assessment, SFR Sylvian fissure ratio, α-HBDH α–hydroxybutyrate dehydrogenase, HSCRP high-sensitivity C-reactive protein, TC total cholesterol, TG total triglyceride, HDLC high density lipoprotein cholesterol, LDLC low density lipoprotein cholesterol, APOA apolipoprotein A, APOB apolipoprotein B.

| Supplemental Table 2. Characteristics of patients divided into dichotomous SFR categorical after Propensity-Score Matching. | | | |
| --- | --- | --- | --- |
| Characteristic Mean(SD) or Median (Q1-Q3) / N(%) | | | P-value |
|  | SFR ≤0.05 (n=71) | SFR >0.05(n=71) |  |
| **Age** | 80.75 ± 4.28 | 84.10 ± 5.06 | <0.0001 |
| **MoCA** | 22.00 ± 5.43 | 18.82 ± 5.93 | 0.0011 |
| **SFR** | 0.03 ± 0.01 | 0.10 ± 0.04 | <0.0001 |
| **Creatinine** | 88.42 ± 36.93 | 89.61 ± 41.14 | 0.8560 |
| **Uric Acid** | 347.27 ± 94.33 | 352.01± 104.83 | 0.7777 |
| **α-HBDH** | 151.09 ± 38.55 | 145.76 ± 26.91 | 0.3411 |
| **HSCRP** | 3.43 ± 4.17 | 5.09 ± 15.58 | 0.3864 |
| **TC** | 4.25 ± 0.80 | 4.08 ± 0.91 | 0.2305 |
| **TG** | 1.25 ± 0.61 | 1.51 ± 0.93 | 0.0553 |
| **HDLC** | 1.24 ± 0.32 | 1.13 ± 0.27 | 0.0300 |
| **LDLC** | 2.50 ± 0.64 | 2.41 ± 0.74 | 0.4417 |
| **APOA** | 1.43 ± 0.27 | 1.41 ± 1.03 | 0.8431 |
| **APOB** | 0.90 ± 0.22 | 0.90 ± 0.25 | 0.9150 |
| **Fazekas scale-No. (%)** | | | 0.0017 |
| 0 | 29 (40.8) | 12 (16.9) |  |
| 1 | 22 (31) | 18 (25.4) |  |
| 2 | 15 (21.1) | 27 (38) |  |
| 3 | 5 (7) | 14 (19.7) |  |
| ***Sex-No. (%)*** | | | 0.2513 |
| male | 49 (69) | 56 (78.9) |  |
| female | 22 (31) | 15 (21.1) |  |
| ***Hypertension -No. (%)*** | | | 0.6220 |
| No | 8 (11.3) | 11 (15.5) |  |
| Yes | 63 (88.7) | 60 (84.5) |  |
| ***Diabetes -No. (%)*** | | | 0.3013 |
| No | 47 (66.2) | 40 (56.3) |  |
| Yes | 24 (33.8) | 31 (43.7) |  |
| ***Coronary Heart Disease-No. (%)*** | | | 0.7069 |
| No | 53 (74.6) | 50 (70.4) |  |
| Yes | 18 (25.4) | 21 (29.6) |  |
| ***Stroke-No. (%)*** | | | 1.0000 |
| No | 60 (84.5) | 59 (83.1) |  |
| Yes | 11 (15.5) | 12 (16.9) |  |

MoCA denotes Montreal Cognitive Assessment, SFR Sylvian fissure ratio, α-HBDH α–hydroxybutyrate dehydrogenase, HSCRP high-sensitivity C-reactive protein, TC total cholesterol, TG total triglyceride, HDLC high density lipoprotein cholesterol, LDLC low density lipoprotein cholesterol, APOA apolipoprotein A, APOB apolipoprotein B.
